# The Contribution of Posttraumatic Stress Disorder and Depression to Insomnia in North Korean Refugee Youth

**DOI:** 10.3389/fpsyt.2019.00211

**Published:** 2019-04-08

**Authors:** Jinme Park, Thomas Elbert, Seog Ju Kim, Jinah Park

**Affiliations:** ^1^Department of Psychology, University of Konstanz, Konstanz, Germany; ^2^Department of Psychiatry, Samsung Medical Center, Sungkyunkwan University School of Medicine, Seoul, South Korea; ^3^Department of Counseling, Kyonggy University, Suwon, South Korea

**Keywords:** multiple trauma, posttraumatic stress disorder, insomnia, depression, North Korean refugee youth

## Abstract

Refugees are exposed to multiple traumatic and stressful events and thereby are at higher risk for developing a variety of psychological sequelae including posttraumatic stress disorder (PTSD). However, the relation of PTSD to other mental health conditions has not been fully revealed in refugee populations. The present study investigated relationships among trauma exposure, PTSD, depression, and insomnia in North Korean refugee youth. Seventy-four refugee youth were assessed for exposure to traumatic events, PTSD, depression, and insomnia symptoms. The results showed high rates of multiple trauma exposures among the refugee youth and high incidences of co-occurring symptoms of PTSD and insomnia in those who have multiple trauma. Furthermore, the overall symptoms and four cluster symptoms of PTSD were strongly correlated with insomnia in addition to depression. In the path model to predict insomnia, PTSD affected insomnia only through depression, indicating that the greater the levels of PTSD suffered, the greater the likelihood for developing sleep problems *via* depression. The present study indicates how sleep problems relate to trauma-related symptoms, i.e., PTSD and depression in refugee populations, and highlights the need for further investigation of the specific relation between sleep problems and trauma-related symptoms for effective evaluation and intervention.

## Introduction

Refugees are frequently exposed to traumatic and stressful events of different types ranging in numbers between 2 and 17 (1–5). The greater the number of traumatic events experienced, the greater the likelihood for developing a variety of psychological sequelae such as posttraumatic stress disorder (PTSD) and depression (5–7) and the smaller the likelihood for remission without treatment (8). A systematic review of the literature on psychiatric prevalence in refugees shows that the heterogeneity of the samples and findings was considerable (9). Importantly, there is no meaningful general, standard prevalence rate for refugees due to the heterogeneity of factors associated with mental health, such as characteristics of trauma unreported on or samples that are difficult to access (10).

Hyperarousal is a defining feature of PTSD and usually includes the inability to obtain a normal sleep. Nightmares contribute to the disturbed sleep patterns. Consequently, the prevalence of insomnia has been found to be elevated in survivors of traumatic stressors. For instance, in a representative population-based sample study, adolescents with a history of childhood adversity were more likely to have insomnia later in life than those who did not report exposure to adversity (11). In addition, experiencing more adversities increased the risk of insomnia, which indicates a dose–response relationship (11). On the other hand, clients with longer pretreatment total sleep time and pretreatment Rapid Eye Movement (REM) sleep duration showed a better treatment outcome (12), whereby Narrative Exposure Therapy (NET) resulted in an increased reduction in sleep latency and a reduction in arousals over time.

Only few studies have examined sleep in refugee children. Hjern et al. (13) found a close link between sleep disturbance and experiences of persecution among Chilean refugee children. Montgomery and Foldspang (14) suggested that the presence of sleep disturbance was predicted by violent exposures and stressful family situations in the refugee children from the Middle East. One study concerning insomnia among adult refugee populations showed high rates of insomnia in North Korean refugees (aged 38 ± 12 years) when compared to the general population (38% vs. 9%) (15). The authors also compared trauma exposure and mental health problems reported by North Korean refugees with insomnia to those reported by North Korean refugees without insomnia and found that North Korean refugees with insomnia reported having experienced a larger number of traumatic events and higher levels of PTSD and depression symptoms. Although these findings suggest that it may be fruitful to investigate insomnia in refugee populations, research on insomnia among these populations is scarce.

PTSD commonly occurs with other mental health outcomes in refugees (16–18). Studies of trauma-affected populations have found the mediation effect of PTSD on mental health outcomes such as depression, substance abuse, physical health, and personality disorder (3, 19, 20). Although research evidence suggests that PTSD may provide a link between trauma exposure and the presence of comorbid mental health symptoms (3, 19, 20), the relationship between PTSD and other mental health conditions has not been fully revealed in refugee populations.

In North Korean refugee adolescents and youth, studies have found high rates of trauma exposure and high levels of consequent psychopathology including PTSD and depression (3, 21, 22–26). North Korean children and adolescents are exposed to multiple traumatic experiences not only in North Korea but also during defection and stay in the third-world country (3, 27). The most commonly reported trauma includes witnessing execution or torture, witnessing traumatic incidents involving family members (death, arrest, etc), experiencing or witnessing violence, chronic malnutrition, hunger-related illnesses, witnessing death from starvation, and incarceration (3, 22, 27). In particular, North Korean female adolescents are likely to face forced marriage, sexual assault, or prostitution by Chinese human traffickers or brokers (27). The association between the level of trauma exposure and the severity of mental health outcomes including PTSD has been found to be robust (3, 22, 24). Kim (3) investigated interrelationships among trauma exposure, PTSD, and comorbid mental health problems in North Korean refugee youth and found that PTSD mediated the relationships between interpersonal trauma and comorbid mental health problems. However, there is a lack of research on insomnia and its relation to trauma exposure and PTSD among North Korean refugee youth.

The aim of the present study was to examine relationships among trauma exposure, PTSD, depression, and insomnia in a sample of North Korean refugee youth. Our first hypothesis was that higher rates of co-occurring symptoms of PTSD and insomnia would be found in those who have experienced a greater number of traumatic events. The second hypothesis was that PTSD would be associated with insomnia as well as depression. Finally, in order to predict insomnia in a single model, we used path analysis to evaluate the third hypothesis that trauma exposure would affect insomnia through PTSD and depression. We assumed that in this sample, depression symptom severity mainly would arise as a consequence of PTSD symptom severity. The paths in the model were predetermined by theoretical assumptions, previous empirical research, and temporal precedence.

## Materials and Methods

### Sample

The sample consisted of 74 refugees (47 females and 27 males) with a mean age of 18.7 years (SD = 2.5; range, 15–29). Of the overall sample, 35 participants (47%) were born in North Korea and 39 participants (53%) were born in China. The youth who were born in China were the children who were born from forced marriages between North Korean women and Chinese men. As these marriages are illegal under Chinese law, the youth were not given citizenship and escaped to South Korea with their mother. Thus, we defined North Korean refugee youth as the youth who were born in North Korea or in China in the present study. The two groups differed with respect to age (*t* = 3.5, *p* = .001) and gender ratio (χ^2^ = 10.7, *df* = 1, *p* = .002). There was no significant difference in the number of trauma types between groups (*t* = 1.4, *p* = .174). With regard to content of the trauma types, we found only one difference between groups. More than half of North Korean youth who were born in North Korea reported having an experience of seeing someone being beaten, shot at, or killed (68.2%), whereas 31.8% of North Korean youth who were born in China had experienced this type of trauma, χ^2^ = 5.908, *df* = 1, *p* = .021.

### Procedure

Participants were recruited from a specialized school for North Korean refugee youth, which offers middle and high school education in South Korea. Anyone who is a North Korean refugee youth can apply to this school and will be assigned to this school selectively according to the decision of the school board. All of the North Korean refugee students (*N = 90*) were invited to participate in this study following the agreement and cooperation of the organization’s leaders. Two researchers administered the questionnaires to participants in their classrooms. Prior to administration, the researcher explained the aim and content of the study, procedure, risks and confidentiality. Participants who volunteered to take part in the study and signed an informed consent formwere then included in this study. For minors an informed consent form signed by their legal guardian was required as well. Participants were asked to complete questionnaires about traumatic experience, post-traumatic stress disorder, depression and insomnia symptoms which were written in Korean. If participants had questions about the questionnaire items, the researcher gave a detailed explanation and clarified the items. During the administration of the study participants were asked to sit away from each other to ensure privacy. Completing the questionnaires required about 40 minutes. The ethical review board of the University of Konstanz approved the present study.

### Instruments

#### Trauma Exposure

The trauma event checklist of the University of California at Los Angeles PTSD Reaction Index for Children/Adolescents for the Diagnostic and Statistical Manual of Mental Disorders, Fifth Edition (DSM-5) (UCLA-PTSD-RI-V) (28) was used for the assessment of exposure to trauma. The checklist contains 14 items covering different traumatic event types. In the present study, the number of different types of traumatic events reported by the participant was defined as the level of exposure to trauma, because PTSD prevalence was most accurately predicted by the number of different traumatic event types experienced (7).

#### PTSD Symptoms

The symptom severity of PTSD was assessed with the UCLA PTSD Reaction Index for C/A DSM-5 (the revised version of the UCLA PTSD RI for DSM-4) (28). The new DSM-5 version consists of 27 items measuring PTSD symptoms corresponding to B, C, D, and E criteria and four additional items assessing dissociative subtype. Each item can be rated from 0 (none) to 4 (most of the time) based on the frequency of symptoms in the past month. In the current study, the severity of PTSD was calculated by summing up the scores of all items of PTSD symptoms (range, 0–80). According to the instructions, the cutoff score for considering PTSD is 38. For the current study, two independent Korean-speaking translators translated the UCLA PTSD Index for DSM-5 (UPID) into Korean, and then another translator, who is bilingual in Korean and English, translated it back into English. A Korean clinical psychologist corrected and confirmed discrepancies. Cronbach’s α for the UCLA PTSD RI in the current sample was .95.

#### Depression Symptoms

The Patient Health Questionnaire-9 (PHQ-9) (29) is a nine-item self-report questionnaire assessing the severity of depression. Each item can be rated from 0 (not at all) to 3 (nearly every day) based on the frequency of symptoms over the last 2 weeks. The sum of all nine items was defined as the severity of depression symptoms (range, 0–27) in the present study. Following the instructions for the PHQ-9 (30, 31), a total score of ≥10 is regarded as indicative of severe depression and the cutoff score for considering treatment. The Korean version of the PHQ-9 has been shown to be an appropriate self-report diagnostic tool for assessing depression (32). Cronbach’s α of the PHQ-9 sum score was .86 in the current sample.

#### Insomnia Symptoms

The Insomnia Severity Inventory (ISI) (33, 34) is a seven-item self-report questionnaire measuring sleep difficulties. Each item can be rated from 0 (no problem) to 4 (very severe problem) based on the nature, severity, and impact of sleep difficulties in the past months. The items assess severity of sleep onset, sleep maintenance, early morning awakening problems, sleep dissatisfaction, inference of sleep difficulties with daytime functioning, noticeability of sleep problems by others, and distress caused by the sleep difficulties. The total sum score is defined as the symptom severity of insomnia (range, 0–28) and interpreted as follows: absence of insomnia (0–7), subthreshold insomnia (8–14), moderate insomnia (15–21), and severe insomnia (22–28). The Korean version of the ISI has been shown to be a clinically useful instrument for assessing the severity of insomnia with good psychometric properties (35). Cronbach’s α in the sample was .83.

### Statistical Analysis

Data analyses were performed using IBM SPSS version 25.0 and AMOS version 24.0. Associations between variables and group differences with regard to sociodemographic variables were examined using either the chi-square test or *t* test. Pearson correlations were used to examine the relations between PTSD, depression, and insomnia symptoms. A path analysis was used to predict insomnia in a single model. Potential impact on results due to symptom overlap between PTSD and depression could be excluded in preliminary analyses. The following indices with recommending values assessed adequacy of model fit: chi-square; root mean square error of approximation (RMSEA), with values below .08 (36); standardized root mean square residual (SRMR), with values less than.05 (37); and goodness-of-fit statistic (GFI) and adjusted goodness-of-fit statistic (AGFI), with values close to .90 or higher. For GFI, a higher cutoff of .95 is recommended, when sample sizes are low (38).

## Results

High rates of multiple trauma exposures were found in our sample. The mean number of traumatic event types was 4.5 (SD = 2.4) with a maximum number of 10. Note that the actual occurrence of traumatic stressors can be much higher, as one type may have been experienced multiple times. The majority (91%) reported having experienced more than two traumatic events and about half (49%) reported having been exposed to more than five different types of traumatic stressors. The five most frequent types were having anyone close to me died (56.8%); seeing a family member being hit, punched, or kicked at home (48.6%); being hit, punched, or kicked at home (40.5%); seeing a dead body (36.5%); and being in an accident (35.1%). Additionally, 68.9% of the total sample reported having other traumatic experiences that are not described in the trauma list of the UPID.

The mean score was 28.43 (SD = 16.69) for PTSD symptoms and 9.81 (SD = 5.90) for depression symptoms. For insomnia symptoms, the mean score was 13.19 (SD = 5.58).

Thirty percent of the sample (*n* = 22) met the cutoff score for PTSD symptoms. Fourteen of them also presented with scores for depression and insomnia above the cutoff.

**Figure 1:**
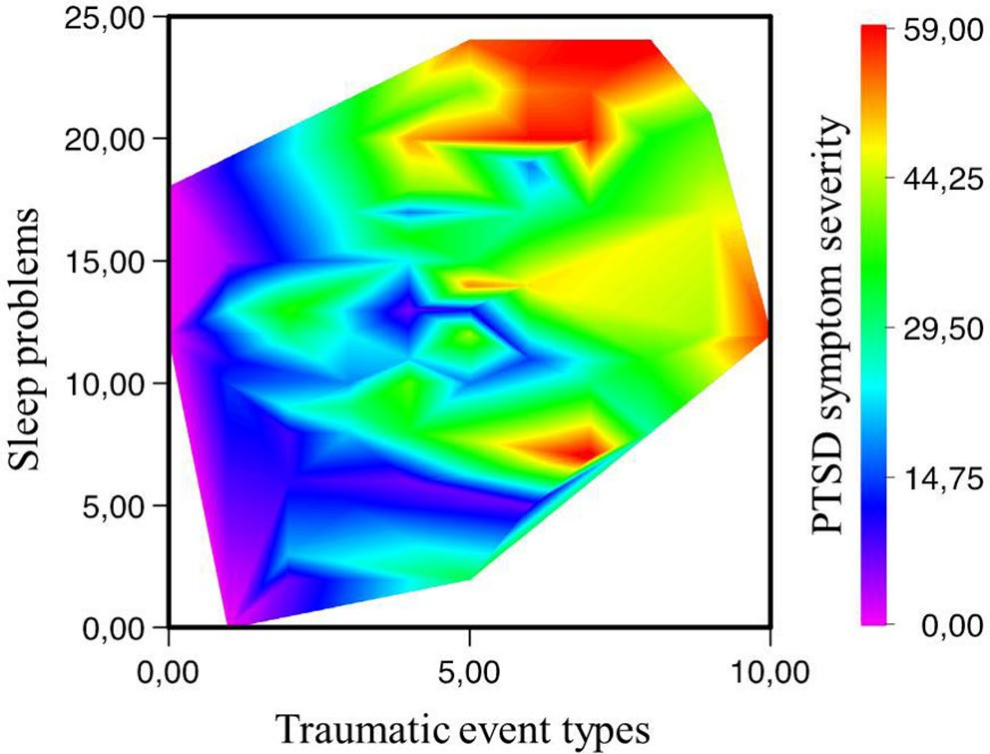
The relationship between the number of traumatic event types and insomnia according to the posttraumatic stress disorder (PTSD) symptom severity. There are no data in the lower right corner (i.e., all respondents who report high numbers of traumatic events also present with sleep problems) and in the upper left corner (sleep problems do not reach extreme values in those with few traumata). *Note*: Sleep problems, the ISI sum score; PTSD symptom severity, the UPID sum score.

**Figure 1** illustrates the high rates of co-occurring symptoms of PTSD and insomnia in those who have multiple trauma.

The correlations among the mental health outcome variables are presented in **Table 1**. The scores of the overall symptoms and the four diagnostic cluster symptoms of PTSD were positively correlated with the score of depression and insomnia symptoms (*p* < .001).

**Table 1:** Correlations between mental health outcomes.

	1	2	3	4	5	6	7
1. Overall PTSD symptoms	—						
2. PTSD intrusion	.88	—					
3. PTSD avoidance	.78	.70	—				
4. PTSD alterations in cognition/emotion	.92	.70	.65	—			
5. PTSD alterations in arousal and reactivity	.87	.68	.55	.72	—		
6. PTSD dissociation symptoms	.69	.62	.41	.70	.55	—	
7. Insomnia symptoms	.56	.42	.39	.53	.55	.43	—
8. Depression symptoms	.77	.62	.54	.78	.65	.59	.59

All correlations are significant at p < .001.

PTSD, posttraumatic stress disorder.

**Figure 2:**
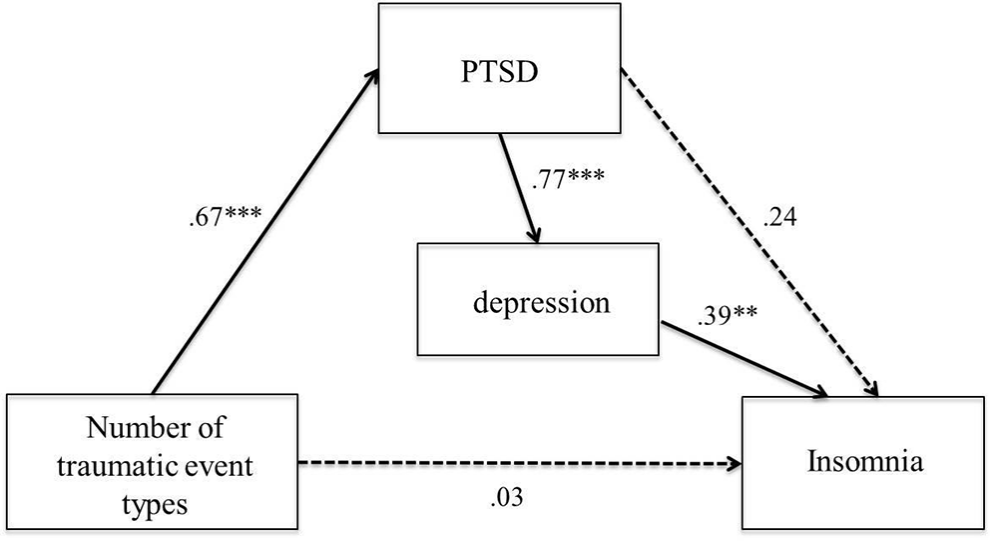
Model representing the associations between trauma exposure, PTSD, depression, and insomnia (*n* = 74). *Note*: Standardized regression weights for all hypothesized paths are presented. Nonsignificant paths are indicated with dotted lines. Error variables are omitted for clarity. ** *p* < .01, *** *p* < .001.

Results of path analysis indicated satisfactory fit to the proposed model, χ^2^ (1, *n* = 74) = .13, *p* = .72; RMSEA = .00; SRMR = .01; GFI = 1.0; AGFI = .99. In the path model, PTSD and depression symptom severity fully mediated the relation between trauma exposure and insomnia symptoms (**Figure 2**). PTSD was significantly associated with depression symptom severity, but not insomnia symptom severity. Trauma exposure was related to PTSD, but not insomnia symptom severity.

## Discussion

The aim of the present study was to examine the associations between trauma exposure, PTSD, depression, and insomnia in a sample of North Korean refugee youth. As expected, the present study revealed high rates of PTSD and currently co-occurring insomnia symptoms among the refugee youth who have experienced a greater number of traumatic events. This is in line with previous findings of high comorbidity rates of PTSD and other mental health symptoms in refugees and war-affected populations (16–17, 18, 39). This is also consistent with studies showing a close relationship between multiple traumatic events and enhanced likelihood of comorbid psychiatric symptoms (40, 41). Moreover, we found positive correlations between PTSD, depression, and insomnia. Specifically, insomnia was strongly related not only to hyperarousal but also to other symptom clusters of PTSD. Results of the path analysis supported the hypothesized associations among trauma exposure, PTSD, depression, and insomnia. The path model indicated that trauma exposure affected insomnia symptom severity through trauma-related symptoms, i.e., PTSD and depression. This confirms the previous finding of a dose–response relationship between exposure to multiple traumatic events and insomnia (11, 15). Our findings are also consistent with studies showing that PTSD provides a link between trauma exposure and depression (3, 16, 17, 20, 39). Furthermore, PTSD affected insomnia symptom severity only through depression in the path model. This suggests that elevated levels of sleep problems in refugee populations may be explained by trauma-related symptoms, especially depression. Therefore, we argue that the presence of depression associated with PTSD increases the risk for developing sleep problems in refugees. In other words, the greater the levels of PTSD suffered, the greater the likelihood for developing sleep problems *via* depression.

Our findings have important clinical implications. First, the present study indicates that the identification of depression associated with PTSD is needed to identify potential candidates that may develop sleep problems later on. In terms of early stage assessment, clinicians and professionals could pay attention to assess depression associated with PTSD to screen individuals with higher risk of sleep problems. Second, in the context of intervention strategy, our findings may be useful to indicate which symptoms should be targeted first to reduce sleep problems of refugees. For example, if sleep problems are related to the presence of depression associated with PTSD, a failure to treat sleep problems may thus be due to persistence of PTSD symptoms. Finally, from the viewpoint of posttreatment evaluation, the improvement of sleep problems may be indicative of good treatment outcomes in the treatment of trauma-related psychological disorders. Weinhold et al. (12) argue that improvement in sleep quality can be considered as an important condition for treatment outcome regarding PTSD symptoms. Considering our finding of the relation between PTSD, depression, and insomnia, treating PTSD may result in improvement in depression symptoms, leading to improved sleep.

There are limitations to the present study. First, the limited sample size may have reduced the accuracy of the statistics. However, values for all indices in the path model represented good model fit with a higher cutoff that is recommended when sample size is small. In addition, our measure was based on retrospective self-report and self-reported symptoms that may include some reporting bias.

The present study extends the existing knowledge and study of sleep problems of refugees and indicates how sleep problems relate to trauma-related symptoms, i.e., PTSD and depression. Our findings call for further investigation of the specific relationship between sleep problems and trauma-related symptoms, leading to more effective evaluation and intervention for refugee populations.

## Ethics Statement

All of the North Korean refugee students (*N* = 90) were invited to participate in this study following the agreement and cooperation of the organization’s leaders. Two researchers administered the questionnaires to participants in their classrooms. Prior to administration, the researcher explained the aim and content of the study, procedure, risks, and confidentiality. Participants who volunteered to take part in the study and signed an informed consent form were then included in this study. For minors, an informed consent form signed by their legal guardian was required as well. The ethical review board of the University of Konstanz approved the present study.

## Author Contributions

All authors developed the study concept and design. JP (1st author) and JP (4th author) conducted data collection under the supervision of SK. JP (1st author) performed the statistical analyses and drafted the paper. TE supervised the data analyses and interpretation of findings. All authors approved the final version of the manuscript.

## Funding

This study was supported by National Research Foundation of Korea (NRF) grant funded by the Korean government (MEST)(No. 2016R1A2B4011561).

## Conflict of Interest Statement

The authors declare that the research was conducted in the absence of any commercial or financial relationships that could be construed as a potential conflict of interest.
