# Corrigendum: The Contribution of Posttraumatic Stress Disorder and Depression to Insomnia in North Korean Refugee Youth

**DOI:** 10.3389/fpsyt.2020.00653

**Published:** 2020-07-10

**Authors:** Jinme Park, Thomas Elbert, Seog Ju Kim, Jinah Park

**Affiliations:** ^1^ Department of Psychology, University of Konstanz, Konstanz, Germany; ^2^ Department of Psychiatry, Samsung Medical Center, Sungkyunkwan University School of Medicine, Seoul, South Korea; ^3^ Department of Counseling, Kyonggy University, Suwon, South Korea

**Keywords:** multiple trauma, posttraumatic stress disorder, insomnia, depression, North Korean refugee youth

In the original article, there was an error. The name of the Ethical Review Committee was not included in the procedure section. Instead of “(edited out for blind review)”, it should be “University of Konstanz”.

A correction has been made to the **Materials and Methods,** subsection **Procedure**:

“Participants were recruited from a specialized school for North Korean refugee youth, which offers middle and high school education in South Korea. Anyone who is a North Korean refugee youth can apply to this school and will be assigned to this school selectively according to the decision of the school board. All of the North Korean refugee students (N = 90) were invited to participate in this study following the agreement and cooperation of the organization’s leaders. Two researchers administered the questionnaires to participants in their classrooms. Prior to administration, the researcher explained the aim and content of the study, procedure, risks and confidentiality. Participants who volunteered to take part in the study and signed an informed consent form were then included in this study. For minors an informed consent form signed by their legal guardian was required as well. Participants were asked to complete questionnaires about traumatic experience, post-traumatic stress disorder, depression and insomnia symptoms which were written in Korean. If participants had questions about the questionnaire items, the researcher gave a detailed explanation and clarified the items. During the administration of the study participants were asked to sit away from each other to ensure privacy. Completing the questionnaires required about 40 minutes. The ethical review board of the University of Konstanz approved the present study.”

The authors apologize for this error and state that this does not change the scientific conclusions of the article in any way. The original article has been updated.

